# Research Trends, Hot Spots, and Prospects for Traditional Chinese Medicine in the Field of Ischemia-Reperfusion Injury

**DOI:** 10.1155/2021/4548367

**Published:** 2021-12-29

**Authors:** Yu Sun, Yuan Cai, Man Xiao, Ming-hai Hu, Guo-qiang Shao, Hui Liu, Bing-bing Shen, Peng-hui Li, Yan-mei Peng

**Affiliations:** ^1^Hunan University of Traditional Chinese Medicine, Changsha, Hunan 410208, China; ^2^Institute of Chinese Materia Medica, Hunan Academy of Chinese Medicine, Changsha, Hunan 410006, China; ^3^Department of Anatomy and Neurobiology, School of Basic Medical Science, Central South University, Changsha, Hunan, China

## Abstract

Ischemia-reperfusion (I/R) injury is one of the most common phenomena in ischemic disease or processes that causes progressive disability or even death. It has a major impact on global public health. Traditional Chinese medicine (TCM) has a long history of application in ischemic diseases and has significant clinical effect. Numerous studies have shown that the formulas or single herbs in TCM have specific roles in regulating oxidative stress, anti-inflammatory, inhibiting cell apoptosis, etc., in I/R injury. We used bibliometrics to quantitatively analyze the global output of publications on TCM in the field of I/R injury published in the period 2001–2021 to identify research hotspots and prospects. We included 446 related documents published in the Web of Science during 2001–2021. Visualization analysis revealed that the number of publications related to TCM in the field of I/R injury has increased year by year, reaching a peak in 2020. China is the country with the largest number of publications. Keywords and literature analyses demonstrated that neuroregeneration is likely one of the research hotspots and future directions of research in the field. Taken together, our findings suggest that although the inherent limitations of bibliometrics may affect the accuracy of the literature-based prediction of research hotspots, the results obtained from the included publications can provide a reference for the study of TCM in the field of I/R injury.

## 1. Introduction

Ischemia-reperfusion (I/R) refers to a pathological state in which the body organs such as the heart [1–3], kidney [4–6], and brain are reperfused and reoxygenated after the blood supply is restricted. Tissue damage caused by ischemia is the main cause of fatal diseases, such as stroke and myocardial infarction caused by coronary atherosclerosis [7]. During the rescue and treatment of ischemic diseases, medical scientists gradually discovered that the main factor that causes damage to tissues is not the ischemia itself but when the blood supply (reperfusion) is suddenly restored after a period of ischemia damage [8, 9]. In traumatic shock, surgery, organ transplantation, burns, frostbite, thrombosis, and other blood circulation disorders, post-ischemia-reperfusion injury will occur. After the blood supply is restored, excessive free radicals attack the cells in the tissues that have regained blood supply. In the ischemic tissues, the synthesis ability of antioxidant enzymes that can scavenge free radicals is hindered, which intensifies the damage of free radicals to the reperfusion tissue after ischemia. Meanwhile, inflammation, oxidative stress, and the accumulation of harmful substances could cause multiple organ damage and microcirculation disorders. This has been proven in many organs [10–13]. However, the current clinical consensus for the treatment of ischemia such as cerebral ischemia is still to use thrombolytic therapy to restore blood supply as soon as possible and reduce the degree of tissue ischemia. Therefore, I/R injuries cannot be avoided. Yet there is a clinical lack of drugs to alleviate ischemia-reperfusion injury. For more than three thousand years, traditional Chinese medicine (TCM) has been widely used in Asian countries, especially China, Japan, and Korea, for I/R injuries. In China, many old and authoritative medical books, such as “*Treatise on Cold Damage* (*Shang Han Lun*),” “*Synopsis of the Golden Chamber* (*Jin Kui Yao Lue*),” “*Important Prescriptions Worth a Thousand Gold for Emergency* (*Qian Jin Fang*),” and “*Correction on Errors in Medical Works* (*Yi Lin Gai Cuo*),” recorded many classical formulas, including Chaihu Jia Longgu Muli Tang, Guizhi Fuling Wan, Huanglian Jiedu Tang, Longdan Xiegan Tang, Shengmai San, and Buyang Huanwu Tang because of the remarkable curative effect of TCM in the treatment of I/R, it has received extensive attention from researchers during its development. Therefore, the research results in the field of TCM for the treatment of I/R have been fruitful. Our research team has been committed to research on TCM. In recent years, we have focused on the treatment of I/R with TCM. We are eager to describe the development of TCM for I/R scientifically, objectively, and quantitatively, so that we can better understand how TCM treats I/R and carry out more in-depth and extensive research. We found that bibliometric analysis can help us achieve this goal. Bibliometric is a widely used method by researchers for quantitative analysis of a specific research field to provide an overview of the research trends encountered in that field. After using mathematical and statistical methods, it generates a broad picture of the field, offering an inner structure pattern. It can reveal the quality, thematic and citation landscape of the literature in the ﬁeld [14]. Through cocitation analysis and citation burst detection, topic clusters, influential contributors, productive institutions, etc., it can offer an accurate description that provides an insight that is beyond the superficial. Meanwhile, benefiting from the development of visualization software, such as VOSviewer and CiteSpace, which is more objective and rigorous and can provide the co-occurrence network diagram.

In this study, we retrieved and collected the research literature on TCM in I/R injury from the Web of Science Core Collection and used VOSviewer and CiteSpace to analyze the literature. The aim of this bibliometric study was to provide a comprehensive overview and inner structure research of TCM in I/R injury. Our work evaluated the efficacy of TCM for any kind of I/R injury. Moreover, innovative approaches such as document cocitation network analysis, research clusters identiﬁcation and analysis, and reference citation bursts detection were performed to offer insights into the research topics and trends evaluation over time from different perspectives. Our analyses will shed new light for investigators, which is helpful for them to plan and manage their scientific work in future research.

## 2. Materials and Methodology

### 2.1. Materials

Literature data of this bibliometrics research were retrieved from the Web of Science Core Collection. Web of Science Core Collection contains several important index types, including Science Citation Index Expanded (SCIE), Social Science Citation Index (SSCI), and Emerging Sources Citation Index (ESCI). In order to make a systematic analysis of I/R injury, we chose citing articles and reviews to make a visual analysis. Search for two terms was carried out, one was “traditional Chinese medicine or Chinese medicinal herbs or traditional Chinese drugs or TCM” and the other was “ischemia-reperfusion injury or ischemia/reperfusion or ischemia reperfusion or ischemia-reperfusion or Ischemia-reperfusion or Ischemic-reperfusion or ODG/R.” The document type was set as article or review, the language as English, and the time span as 2001–2021. The search was completed on May 7, 2021.

After checking, the 612 documents were retrieved from the core collections of the Web of Science. We screened and inspected it with an excel table. Through analyzing the titles, abstracts, and even some full-texts, we removed the documents whose content had nothing to do with the treatment of I/R injury with traditional Chinese medicine. Finally, the remaining 446 documents were used for further bibliometric analysis.

### 2.2. Methodology

Bibliometric analysis and network visualization were performed with VOSviewer (version 1.6.14, developed by Nees Jan van Eck and Ludo Waltman, the Netherlands) and CiteSpace (version 5.6.R4, developed by Chen Chaomei, England). Microsoft Excel 2010 was used to perform the distribution of publication years. Gunn map (http://lert.co.nz/map/) online world map was used to make the distribution of countries and regions.

## 3. Results

### 3.1. Distribution of Publications by Year and Organ

**Figure 1:**
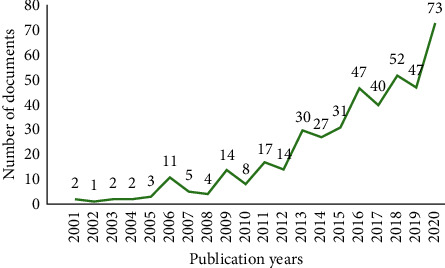
Distribution of publication years from 2001 to 2020.

**Figure 2:**
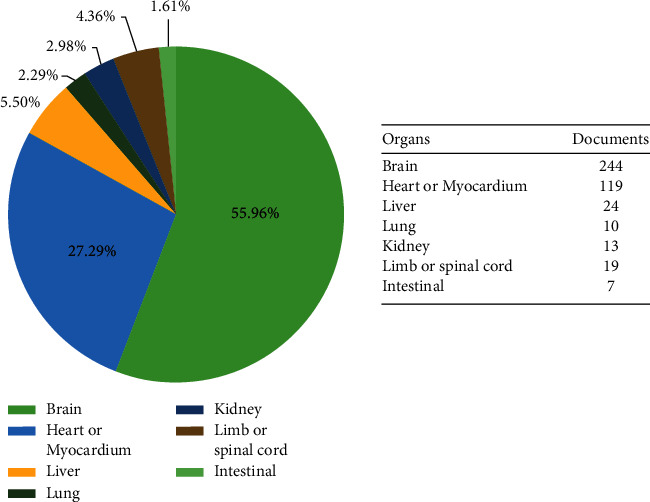
Number of documents of I/R injury in different organs.

We got a total of 612 documents, excluding the duplicates and irrelevant ones, leaving 446. The number of documents in each year is shown in Figure 1. As shown in the figure, the literature on I/R injury from 2001 to 2020 has an overall upward trend, reaching the highest in 2020 with a total of 73 articles published. The TCM on I/R injury is attracting more and more attention from investigators, indicating that it will gradually become a research hotspot even into the future. We have performed a statistical analysis of the I/R injury research of different organs involved in the literature. Except for 10 reviews that cannot be clearly divided, the statistics on the number of documents on each organ are shown in Figure 2. It is clear that the research of I/R injury is mainly cerebral I/R injury, followed by heart and myocardial I/R injury.

### 3.2. Countries and Regions

**Figure 3:**
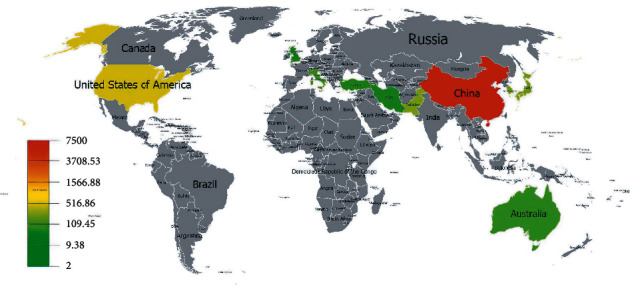
Spatial distribution of the citations of global publications.

According to statistical analysis, a total of 446 documents have been published by research groups from 26 countries and regions. We used the total number of citations of documents in each country and region to make a graph that is convenient for viewing, as shown in Figure 3. In addition, we have counted the top 10 countries and regions with the most published documents, as shown in Table 1. It can be seen from the chart that Chinese research on the treatment of I/R injury with TCM is far ahead in terms of the number of publications and the number of citations. It is worth noting that although the second-ranked United States only published 27 documents between 2001 and 2021, its citations reached 462, with an average citation rate of 5.84%, which is higher than the Chinese figure.

### 3.3. Organizations

**Figure 4:**
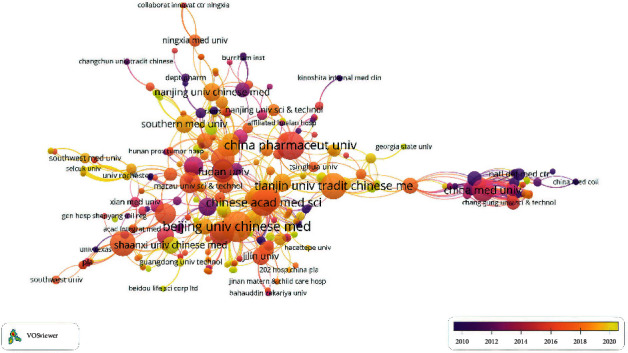
Visualization of organized documents data.

According to VOSviewer analysis, 446 documents were published by 478 different organizations. After excluding 105 disjointed organizations, the network diagram of the remaining 373 organizations is shown in Figure 4. At the same time, according to the number of output documents, we also list the top ten institutions, as shown in Table 2. From the chart, the top ten institutions are all from China, which shows that China's research on the treatment of I/R injury with TCM is dominant. Among them, Beijing University of Chinese Medicine is the most prolific institution (*n* = 26), and China Pharmaceutical University is the most cited institution (*n* = 494). In addition, the average publication year of Beijing University of TCM is 2017, and the corresponding node color is orange, indicating that Beijing University of TCM has the highest document output rate in 2017.

### 3.4. Journals

**Figure 5:**
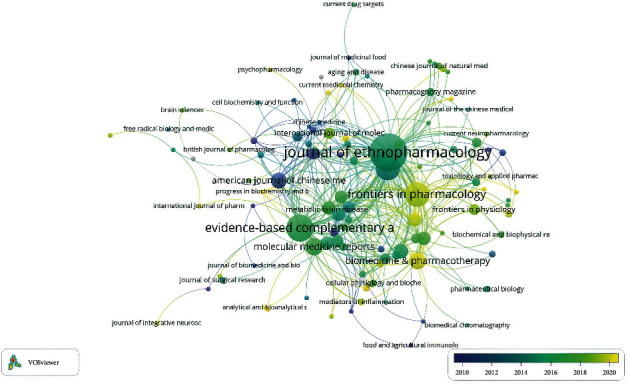
Visualization of journals documents data.

By analyzing the source of publications, it helps to find core journals. Based on data analysis, documents related to TCM in I/R injury published from 2001 to 2021 were distributed in 141 different journals. Excluding 30 journals unrelated to other journals, the relationship network diagram of the remaining 111 journals is shown in Figure 5. Meanwhile, the top ten journals and their 2020 impact factors are shown in Table 3. It can be seen from Table 3 that the *Journal of Ethnopharmacology* has the most publications on TCM in I/R injury, including 61 documents. The 2020 impact factor of the *Journal of Ethnopharmacology* is 3.69. Although *Pharmacological Research* has an impact factor of 5.893 in 2020, it has just recorded 8 documents. So, judging from the number of publications and impact factor of journals, the *Journal of Ethnopharmacology* may be the most influential journal on the treatment of I/R injury with TCM.

### 3.5. Authors

**Figure 6:**
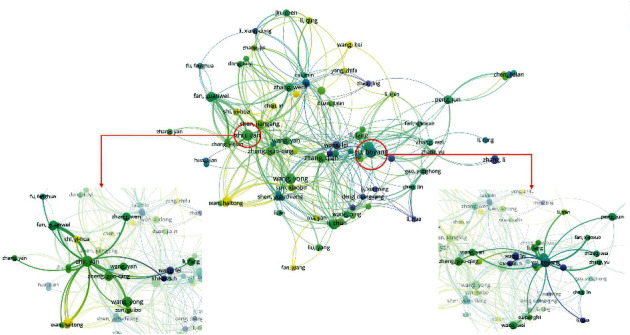
Visualization of authors documents data.

A total of 2621 authors were joined in 446 documents. According to the author's literature number and the number of citations, we can get the core authors in the field of TCM in I/R injury. The evaluation criteria of core authors included the number of published documents and total citations.  Table 4 lists the specific parameters, and the author relationship is shown in Figure 6. Zhu Yan and Yu Boyang both have 10 documents, while the number of citations of Yu Boyang (*n* = 215) is higher than that of Zhu Yan (*n* = 196). In addition, from the visualization map in Figure 4, we can observe that the two authors are not related. After comparison, it is found that Zhu Yan has 3 documents related to the brain and 7 documents related to the heart and myocardium, while Yu and Boyang have 7 documents related to the brain and 3 documents related to the heart and myocardium. It can be concluded that Zhu Yan and his team are mainly dedicated to the study of heart and myocardial I/R injuries, and Yu Boyang and their team are mainly dedicated to the study of cerebral I/R injuries. In addition, there are many emerging research teams involved in the emergence of I/R injuries, indicating that I/R injuries are still a hot spot.

### 3.6. Keywords

**Figure 7:**
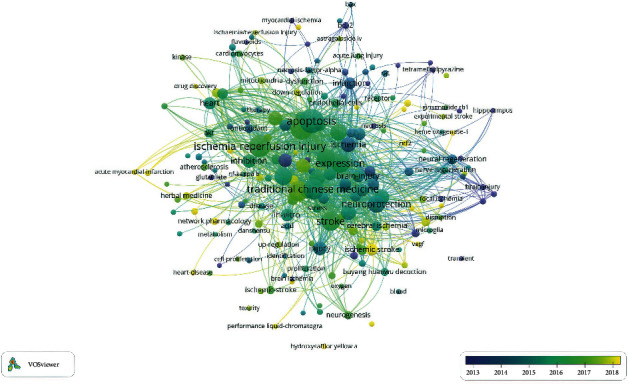
Visualization of keywords data.

**Figure 8:**
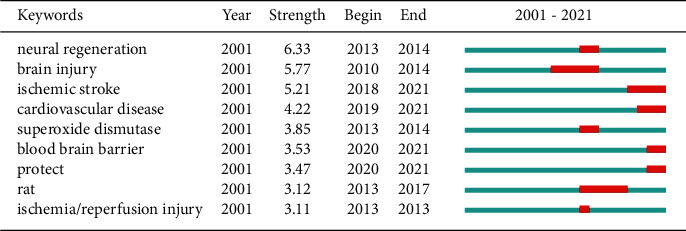
Top 9 keywords with the strongest citation bursts.

Keywords are the core vocabulary of an article and appear frequently. Searching and analyzing based on keywords is a quick and direct way to understand this article. There are a total of 2311 keywords in the 446 documents. After selecting the first 200 words, excluding words such as “ischemia-reperfusion injury,” which would obviously be high-frequency words, the remaining words network visualization map shows the co-occurrence relations as shown in Figure 7. The size of the circle indicates the occurrence of keywords. In addition, we used Table 5 to list the top 10 keywords. As shown in Table 5, the high-frequency keywords are apoptosis, oxidative stress, and stroke, indicating that the molecular mechanisms of stroke in I/R injury treated with TCM are a hotspot. At the same time, we used CiteSpace to make a burst map, as shown in Figure 8, strength represents the intensity of the burst, and the red bar represents the duration of the hot spot. Among them, the intensity value of nerve regeneration is the highest, but the burst time is shorter, and it was only a research hotspot in 2013–2014. According to the figure, in the past three years, the protection of ischemic stroke, cerebral infarction, and other cardiovascular and cerebrovascular diseases has been the current research hotspot of TMC in the treatment of I/R injury.

### 3.7. Citations

**Figure 9:**
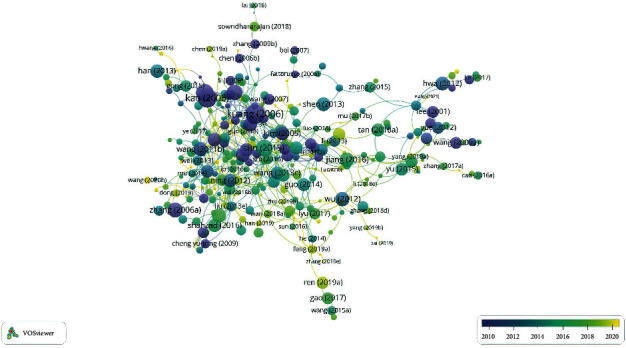
Visualization of citations data.

According to the literature citation analysis of documents, which reflects the number of citation times of the literature, the following observations are drawn: Among the 446 documents, “*Pinocembrin: A Novel Natural Compound with Versatile Pharmacological and Biological Activities*” ranked first, published by Rasul and Azhar in 2013 and cited 142 times. However, in our visual analysis process, we found that this document does not form a network with other articles. That is to say, although this document has the highest number of citations, other documents and this document are not mutually cited, so we eliminated it.  Table 6 lists the top 10 documents with the remaining number of citations. The mutual network diagram of the remaining documents is shown in Figure 9. It can be seen from the chart that the “*Neuroprotective role of Z-ligustilide against forebrain ischemic injury in ICR mice*” published by Kuang X in 2006 is the most cited document on the net. The second and third places are “*Neuroprotection by tetramethylpyrazine against ischemic brain injury in rats*” published by Kao TK in 2006 and “*Tetramethylpyrazine reduces ischemic brain injury in rats*” published by Liao SL in 2004. Obviously, these three articles are documents related to TCM and cerebral I/R injury and all have a certain relationship with neuroprotection, which further confirms that the treatment of cerebral I/R injury by TCM through neuroprotection is the main concern.

## 4. Discussions

During the preceding two decades covered by this research, the number of annual publications increased gradually and reached a peak in 2020. The curve indicated that TCM on I/R injury has drawn more and more attention, and it will continue to be a research hotspot. Moreover, according to the data analysis of document types, we found that most of them were original articles, and only a few were reviews. The reason behind this phenomenon is that there is a continuing requirement for novel investigation at this stage.

Taken together, 2311 keywords were retrieved from all of the documents. The statistical analysis of keywords suggests that we need to regard the statistical results of word frequency of keywords as a net structure, as shown in Figure 7. According to the statistical results of key molecules, apoptosis is the most common form of cell death, indicating that apoptosis has received more attention from researchers. Furthermore, the number of studies on other mechanisms (including oxidative stress, inflammation, and neuroprotection) and stroke ranked high is greater than those on other types, and the total link strength is also high. From the perspective of bibliometrics, this phenomenon shows that these molecules or diseases are frequently studied as research subjects. In addition, we listed the major molecular mechanisms of some classic single herbs and formulas in Table 7. These results can be used as a reference when investigators select their starting point in TCM on I/R injury.

By analyzing the distribution of publication sources, judging from the number of publications and the impact factor of journals, *The Journal of Ethnopharmacology* might be the most influential journal. As we know, *the Journal of Ethnopharmacology* is dedicated to the exchange of information and understandings about people's use of plants, animals, and microorganisms and their biological and pharmacological effects. This journal is very friendly to research articles on ethnic medicine including prescription. Ranked second is *Evidence-Based Complementary and Alternative Medicine*, the referred journal is an international, peer-reviewed journal that seeks to apply scientific rigor to the study of complementary and alternative medicine modalities, particularly traditional Asian healing systems. Precisely, TCM has been used for a long time in Asia, with a very strong history of traditional application and distinctive features, which is very much in line with the taste of the journal. Tied for third is *Neural Regeneration Research* and *Frontiers in Pharmacology*. *Neural Regeneration Research* mainly focuses on neurological research. Compared with *the Journal of Ethnopharmacology* and *Evidence-Based Complementary and Alternative Medicine*, it is more targeted for research related to stroke and other neurological injury diseases, while *Frontiers in Pharmacology* is more comprehensive and includes the latest research in pharmacology. Grasping and paying attention to the core journals in this field will not only help us quickly understand the research progress in this field. On the other hand, we can also consider submitting articles to these journals for our own research results in this field.

**Figure 10:**
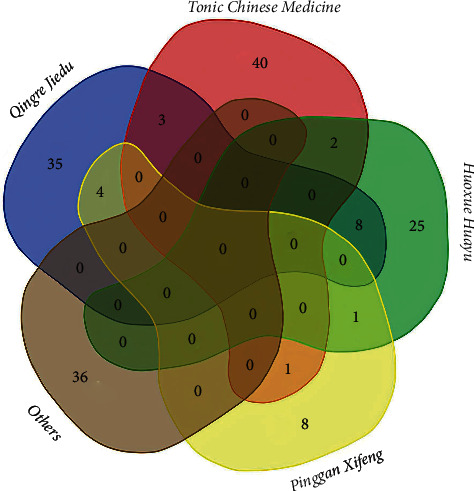
Classification of five major types of Chinese medicine.

As we know, TCM has its own unique theory. In the theory of TCM, I/R injury is attributed to *Qi* and blood deﬁciency, blood stasis syndrome, toxic heat (Chinese name: *Redu*) accumulation, and wind moving within the liver (Chinese name: *Ganfeng Neidong*). According to our quantitative analysis, as shown in Figure 10, by sorting out the types of TCM, the research on the treatment of I/R injury with TCM mainly focuses on the following categories:

1. Tonic Chinese medicine. As we all know, *Panax ginseng* C. A. Mey. (Chinese name: *Renshen*), *Astragalus* membranaceus (Chinese name: *Huangqi*), *Salvia miltiorrhiza* Bge. (Chinese name: *Danshen*), etc., are classic and effective tonics in Chinese medicine. Liang et al. found that Danshen can improve liver I/R injury through antioxidation, promote microcirculation, and reduce Kupffer cell activation [25]. In a rat MCAO model treated with *Astragalus* injection, it was found that *Astragalus* injection can downregulate the expression of the JNK3 gene after cerebral I/R injury in rats, inhibit neuronal apoptosis, reduce infarct volume, and improve neurobehavioral function [26]. Researchers used the H9C2 cardiomyocyte hypoxia/glucose reoxygenation model to explore the effect of ginseng polysaccharides on myocardial I/R injury. The results showed that ginseng polysaccharides can maintain the function of myocardial mitochondria, thereby inhibiting myocardial ischemia. Cell apoptosis caused by reperfusion also increases the expression of glucocorticoid receptors and estrogen receptors, which in turn mediates the activation of the RISK pathway and the endothelial nitric oxide synthase-dependent mechanism to resist reperfusion injury [27].
2. Activating blood and removing blood stasis (Chinese name: *Huoxue Huayu*). TCM for promoting blood circulation and removing blood stasis is widely used in vascular diseases, almost all prescriptions contained. Studies have found that this type of Chinese medicine has miraculous effects. *Panax notoginseng* ( Burk.) Chen (Chinese name: *Sanqi*), as a typical TCM for promoting blood circulation and removing blood stasis, studies found that *Panax notoginseng* saponins can directly downregulate the overexpression of proinflammatory factors like interleukin-1*β* and tumor necrosis factor-*α*, and at the same time upregulate the expression of anti-inflammatory factors like interleukin-10 in the core area of cerebral infarction, thereby preventing permanent nerve damage in rats after MCAO. At the same time, the researchers also combined with the SH-SY5Y cell model of OGD/R injury and found that notoginseng total saponins can also provide neuroprotection by inhibiting the overexpression of *NgR1*, *RhoA*, and *ROCK* [28, 29].
3. Antipyretic and antidote (Chinese name: *Qingre Jiedu*). As early as the Tang Dynasty, Sun Simiao's “*Qianjin Yifang*” was recorded on the treatment of strokes with heat clearing and detoxifying methods. Modern studies have shown that the treatment of antipyretics and antidotes has significant advantages over the control group in terms of improving neurological function and anti-inflammatory effects. In addition, studies showed that geniposide may inhibit H/R-induced cardiomyocyte apoptosis by activating *GLP-1R* and *PI3K/AKT* signaling pathways and reversing mitochondrial dysfunction [30].
4. To calm the liver and relieve wind (Chinese name: *Pinggan Xifeng*). The famous document “*Su wen*” recorded that “*all winds are dizzy, all belong to the liver.*” In the theory of TCM, many endovascular diseases such as stroke and hypertension are caused by the hyperactivity of *Ganyang shangkang*. Studies showed that the phenolic substances in Gastrodia elata Bl. (Chinese name: *Tianma*) can stimulate the endogenous antioxidant response of astrocytes and neurons and promote reactive astrocytes to provide antioxidants and nerves to neurons, thereby reducing neuronal damage [31].

There are many forms of TCM for the treatment of I/R injuries. Clinically, it is mainly used for single herbs and prescriptions. To learn more about the most researched single herbs and prescriptions, we listed the top ten most frequently prescribed single herbs in Table 8. According to the statistics, *Danshen* and *Buyang Huanwu Decotion* ranked first in single herbs and prescriptions, respectively. As we know, *Danshen* is a classical medicine for activating blood and removing blood stasis and belongs to the *Huoxue Huayu* category. *Buyang Huanwu Decotion* is recorded in “*Correction on Errors in Medical Works* (*Yi Lin Gai Cuo*)”in the *Qing* Dynasty. It has a miraculous effect on the clinical sequelae of cerebrovascular accidents, as well as hemiplegia and paraplegia caused by other reasons. *Buyang Huanwu Decotion* is mainly composed of *Huangqi*, *Chuangxiong*, *Honghua*, etc., *Huoxue Huayu* category medicines. This concept is consistent with the current clinical concept of restoring blood supply as soon as possible for ischemic diseases. We further disassembled the prescription and analyzed it together with the single herbs and divided it according to the theory of TCM. As shown in Table 9, the *Qingre Jiedu* is the most widely used, followed by *tonic Chinese medicine*. The abovementioned results indicate that the first consideration for TCM in the treatment of ischemic diseases is also to restore blood supply. However, while supplying blood, the use of *Qingre Jiedu* medicines can reduce the toxic and side effects caused by reperfusion. In addition, *tonic Chinese medicine* could comprehensively strengthen the body's resistance through multiple channels and multiple targets working together to achieve curative effects. This is also the reason for the remarkable curative effect and charm of TCM.

## 5. Conclusions

This study is the first bibliometric study using visual analysis software to analyze publications of TCM in the treatment of I/R injury. Through system statistics and summary, we found TCM has a significant effect on I/R injury. Furthermore, this research field has received widespread attention from researchers around the world. However, how to scientifically and rationally evaluate the efficacy of TCM in the treatment of I/R injury, clarify its specific mechanism, and how to deeply explore the advantages of TCM in the treatment of I/R injury are the key issues that need to be strengthened in the future. Overall, this study provides insight into the trends and characteristics of TCM in the treatment of I/R injury, and should provide a helpful reference for further in-depth research.

## Figures and Tables

**Table 1:** Top 10 most productive countries/regions with publications on TCM in I/R injury.

Rank	Country/region	Documents	Citations	Total link strength
1^st^	China	424	7463	52
2^nd^	USA	27	462	37
3^rd^	South Korea	9	234	3
4^th^	Japan	7	228	5
5^th^	Australia	5	90	6
6^th^	Italy	4	115	14
7^th^	England	4	52	4
8^th^	Pakistan	2	254	4
9^th^	Iran	2	56	10
10^th^	Turkey	2	64	10

**Table 2:** Top 10 most productive organizations.

Rank	Organizations	Documents	Citations	Total link strength
1^st^	Beijing University of Chinese Medicine	26	310	35
2^nd^	China Pharmaceutical University	22	494	17
3^rd^	Chinese Academy of Medical Sciences	19	310	57
4^th^	Tianjin University of Traditional Chinese Medicine	19	270	28
5^th^	Fourth Military Medical University	18	242	23
6^th^	Capital Medical University	16	412	32
7^th^	Peking Union Medical College	15	265	48
8^th^	China Academy of Chinese Medical Sciences	15	175	32
9^th^	Shanghai University of Traditional Chinese Medicine	15	232	21
10^th^	Zhejiang University	15	167	21

**Table 3:** Top 10 most productive journals.

Rank	Journals	Documents	2020 impact factor
1^st^	Journal of Ethnopharmacology	61	3.69
2^nd^	Evidence-Based Complementary and Alternative Medicine	31	1.813
3^rd^	Frontiers in Pharmacology	26	4.225
4^th^	Neural Regeneration Research	26	3.171
5^th^	Molecular Medicine Reports	13	2.1
6^th^	Biomedicine and Pharmacotherapy	12	4.545
7^th^	Am J Chinese Med	11	3.498
8^th^	BMC Complementary and Alternative Medicine	9	2.833
9^th^	Pharmacological Research	8	5.893
10^th^	PLOS One	8	2.74

**Table 4:** Top 10 most productive authors.

Rank	Authors	Documents	Citations
1^st^	Yu Boyang	10	215
2^nd^	Zhu Yan	10	196
3^rd^	Wang Yong	9	96
4^th^	Zhang Qian	8	112
5^th^	Kou Junping	8	174
6^th^	Zheng Guoqing	7	117
7^th^	Li Chun	6	76
8^th^	Wang Wei	6	80
9^th^	Fan Guanwei	6	129
10^th^	Li Fang	6	58

**Table 5:** Top 10 most occurring words.

Rank	Keywords	Occurrences	Total link strength
1^st^	Apoptosis	114	835
2^nd^	Oxidative stress	95	681
3^rd^	Stroke	71	497
4^th^	Expression	69	509
5^th^	Activation	60	435
6^th^	Neuroprotection	57	460
7^th^	Inflammation	53	364
8^th^	Mechanisms	47	345
9^th^	Brain	45	336
10^th^	Artery occlusion	43	341

**Table 6:** Top 10 most cited documents.

Rank	Title (year)	Citations
1^st^	Neuroprotective role of Z-ligustilide against forebrain ischemic injury in ICR mice (2006)	130
2^nd^	Neuroprotection by tetramethylpyrazine against ischemic brain injury in rats (2006)	120
3^rd^	Tetramethylpyrazine reduces ischemic brain injury in rats (2004)	103
4^th^	Ameliorating effects of traditional Chinese medicine preparation, Chinese materia medica and active compounds on ischemia/reperfusion-induced cerebral microcirculatory disturbances and neuron damage (2015)	96
5^th^	Neuroprotective effect of morroniside on focal cerebral ischemia in rats (2010)	79
6^th^	Pharmacological actions and therapeutic applications of salvia miltiorrhiza depside salt and its active components (2012)	70
7^th^	Geniposide prevents hypoxia/reoxygenation-induced apoptosis in H9c2 cells: improvement of mitochondrial dysfunction and activation of GLP-1R and the PI3K/AKT signaling pathway (2016)	68
8^th^	Anti-aging implications of astragalus membranaceus (huangqi): a well-known Chinese tonic (2017)	67
9^th^	Total saponins of *Panax notoginseng* modulate the expression of caspases and attenuate apoptosis in rats following focal cerebral ischemia-reperfusion (2009)	66
10^th^	Neuroprotective herbs for stroke therapy in traditional eastern medicine (2005)	64

**Table 7:** Examples of typical mechanisms of TCM against I/R injury.

Mechanism	TCM	Model	Specific mechanism	Ref.
Antioxidant stress	Xueshuantong injection	MCAO	Nrf2, HO-1, and NQO1↑, activate the Nrf2-VEGF pathway to promote angiogenesis and antioxidant effects	[15]
	Salvia	MCAO	By scavenging free radical activity	[16]
Anti-inflammatory	Guizhi-Fuling capsules	MCAO	IL-1*β* and TNF-*α*↓, IL-10 and IL-10R↑	[17]
	Qingkailing	OGD/R	TNF-*α*,COX-2, iNOS, and p-p38↓	[18]
Antiapoptosis	Tongxinluo	MCAO	Effectively protects ischemia-reperfusion injury through the Cx43/calpain II/bax/caspase-3 pathway and reduces cell death	[19]
	Tongxinluo	MCAO	Mediated by activating the PI3K/Akt pathway	[20]
	Emodin	OGD/R	Induces the expression of Bcl-2 and GLT-1 through the ERK-1/2 signaling pathway, inhibits neuronal apoptosis and reactive oxygen generation, and reduces glutamate toxicity	[21]
	Qingda granule	OGD/R	lncRNA GAS5, bax, caspase-3↓, miR-137, and Bcl-2 ↓	[22]
Neuroprotection/neuroregeneration	Shouwu Yizhi decoction	MCAO	By upregulating the expression of miR210 induced by VEGFA, the notch pathway is activated to promote angiogenesis	[23]
	Huatuo Zaizao pill	MCAO	BDNF↑, improves the neurogenesis level of cerebral ischemic animals	[24]

**Table 8:** Top ten single herb in the literature.

Sort	Chinese name	Latin name	Documents
1	Danshen	*Salviae miltiorrhizae* radix et rhizoma	21
2	Renshen	*Ginseng* radix et rhizoma	16
3	Huangqin	*Scutellariae* radix	12
4	Honghua	*Carthami* flos	9
5	Chuanxiaong	*Chuanxiong* rhizoma	7
5	Gegen	*Puerariae lobatae* radix	7
5	Sanqi	*Notoginseng* radix et rhizoma	7
6	Wuweizi	*Schisandrae chinensis* fructus	5
7	Danggui	*Angelicae sinensis* radix	4
7	Huangqi	*Astragali* radix	4

**Table 9:** Top ten prescriptions in literature.

Sort	Compound name	Compound components	Documents
1	Buyang Huanwu decoction	Huangqi, Danggui, Chishao, Dilong, Chuanxiong, Honghua, Taoren	15
2	Tongxinluo	Renshen, Shuizhi, Quanxie, Chishao, Chantui, Tubiechong, Wugong, Tanxiang, Jiangxiang, Ruxiang, Suanzaoren, Bingpian	13
3	Danhong injection	Danshen, Honghua	8
4	Huanglian Jiedu decoction	Huanglian, Huangqin, Huangbo, Zhizi	6
5	Shenfu injection	Hongshen, Heishunpian	4
5	Shengmai san	Renshen, Maidong, Wuweizi	4
6	Taohong Siwu soup	Danggui, Shudi, Chuanxiong, Baishao, Taoren, Honghua, Niuhuang, Shuiniujiao, Shexiang, Zhenzhu, Zhusha	3
6	Angong Niuhuang wan	Xionghuang, Huanglian, Huangqin, Zhizi, Yujin, Bingian	3
6	Gualou Guizhi soup	Gualou, Guizhi, Baishao, Gancao, Shengjiang, Dazao	3
6	Yiqi Fumai injection	Hongshen, Maidong, Wuweizi	3

## Data Availability

The data used to support the findings of this study are included within the article.

## Abbreviations

I/R: Ischemia-reperfusion
TCM: Traditional Chinese medicine.

## Supplementary Materials

Supplementary Materials Graphical abstract to describe the frame and elaborate the theme of the article Figure S1.
