# Supplementary material for: Research Trends, Hot Spots, and Prospects for Traditional Chinese Medicine in the Field of Ischemia-Reperfusion Injury
Source: Evid Based Complement Alternat Med. 2021 Dec 29;2021:4548367. doi: 10.1155/2021/4548367 (PMC8731293; doi:10.1155/2021/4548367)
Supplement: Supplementary Materials — Graphical abstract to describe the frame and elaborate the theme of the article Figure S1. [file 4548367.f1.pdf]

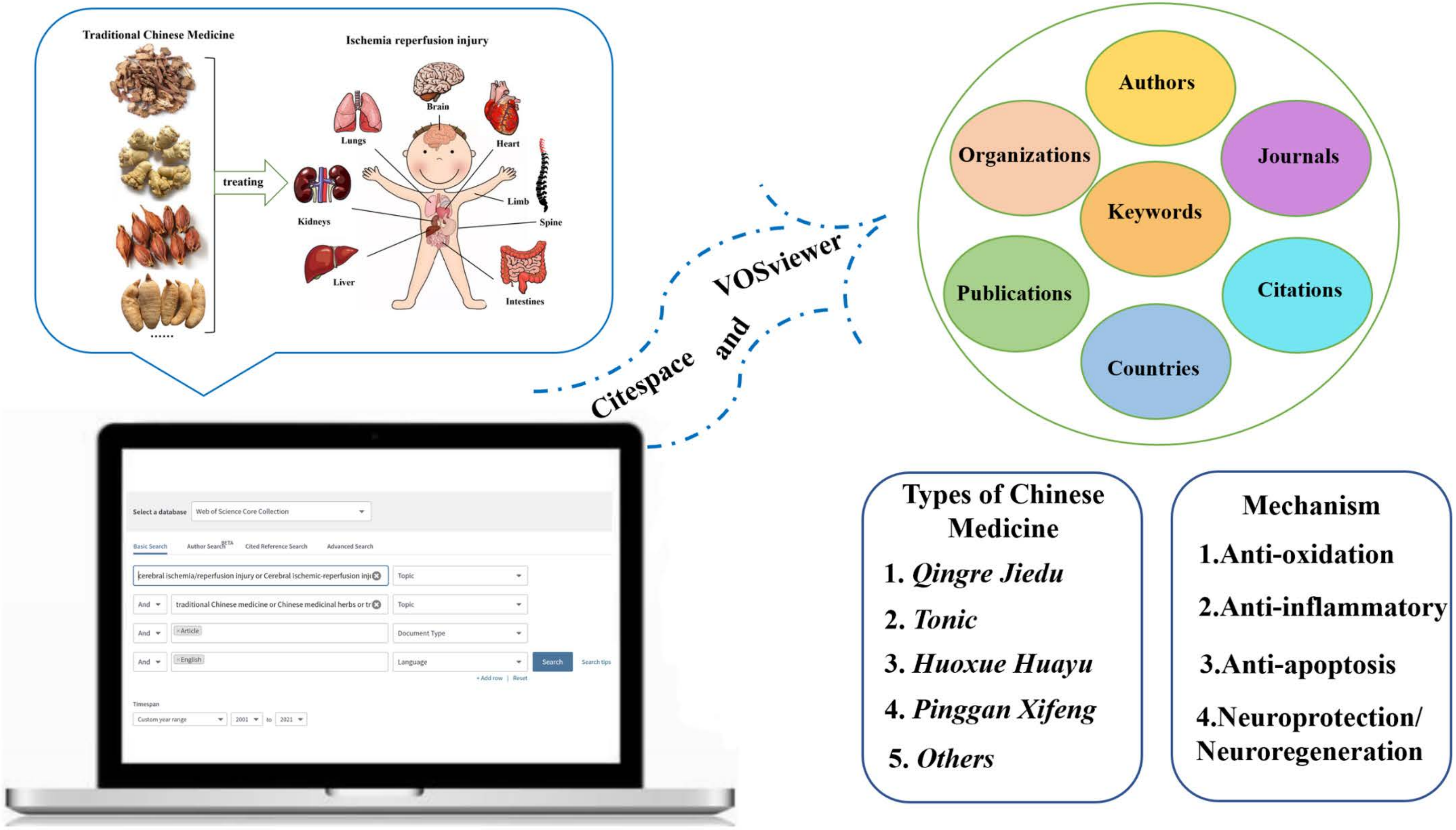
Traditional Chinese Medicine
Ischemia reperfusion injury
treating
Brain
Lungs
Heart
Kidneys
Limb
Spine
Liver
Intestines
......
Select a database
Web of Science Core Collection
Basic Search
Author Search
Cited Reference Search
Advanced Search
Search
Citespace and VOSviewer
Authors
Organizations
Journals
Keywords
Publications
Citations
Countries
Types of Chinese Medicine
1. Qingre Jiedu
2. Tonic
3. Huoxue Huayu
4. Pinggan Xifeng
5. Others
Mechanism
1.Anti-oxidation
2.Anti-inflammatory
3.Anti-apoptosis
4.Neuroprotection/ Neuroregeneration
